# Environmental stress shapes persistence-like phenotypes and genomic changes in *Escherichia coli* and *Morganella morganii:* an exploratory study

**DOI:** 10.3389/fmicb.2026.1749211

**Published:** 2026-03-17

**Authors:** Tosin Yetunde Senbadejo, Samuel Ntiamoah Osei, Elizabeth W. Bugase, Christina R. Bourne, Abiola Isawumi

**Affiliations:** 1 West African Centre for Cell Biology of Infectious Pathogens (WACCBIP), Department of Biochemistry, University of Ghana, Ghana, Accra; 2 AMR Research Group, West African Centre for Cell Biology of Infectious Pathogens, College of Basic and Applied Science, University of Ghana, Legon Accra, Legon, Ghana; 3 Department of Chemistry and Biochemistry, University of Oklahoma, Norman, OK, United States

**Keywords:** *Escherichia coli*, *Morganella morganii*, persistence, stress adaptation, temperature and PH, antibiotic tolerance

## Abstract

**Introduction:**

Enterobacterales, including *E. coli* and *M. morganii*, employ adaptive mechanisms to withstand environmental and host-related stressors, including extreme temperatures, osmotic pressure changes, acidic conditions, and antibiotic pressures. Survival in these conditions can consequently enhance their antibiotic tolerance and persistence. Bacterial persistence contributes to chronic infections and antibiotic treatment failure. This exploratory study investigates the impact of stress conditions (pH, temperature, osmotic, and antibiotic stress) on persistence and genomic adaptations in *E. coli* and *M. morganii*.

**Methods:**

Clinical and environmental *M. morganii* and *E. coli* strains from Ghanaian tertiary hospitals were exposed to extreme temperatures (cold ~4 °C, and heat ~45 °C), extreme pH (pH 3, pH 9, pH 10), and hyperosmolarity (1.71M NaCl). Growth kinetics were monitored by OD600 and CFU determinations, and persister-like cell formation was assessed using time-kill assays at 2 × MIC antibiotic conditions. Genomic changes associated with stress recovery including both adaptive mutations and enrichment of pre-existing variants were captured using comparative whole genome sequencing (WGS) of stress-recovered isolates to parental strains.

**Results:**

Strains exhibited variation in growth kinetics under different stress conditions compared to controls. Temperature, pH, and osmotic stress each affected bacterial growth to varying degrees. Heat stress in particular promoted increased persister-like cell formation in *E. coli* (with strain-specific differences) and, to a lesser extent, in *M. morganii* upon exposure to meropenem as observed in the isolates examined in this study. WGS analysis revealed that all four studied strains harbored virulence and resistance genes, with missense mutations detected in stress-recovered variants. Most of the mutated genes encode proteins that may play key roles in metabolic processes, transport functions and transcriptional regulations.

**Discussion:**

This exploratory study suggests that environmental stress drives both phenotypic and genotypic changes, and that these enhance subsequent survival upon challenge with antibiotics. These adaptive responses may contribute to antibiotic tolerance and chronic infection, emphasizing the need for therapeutic strategies targeting stress response pathways.

## Graphical Abstract

**Figure:**
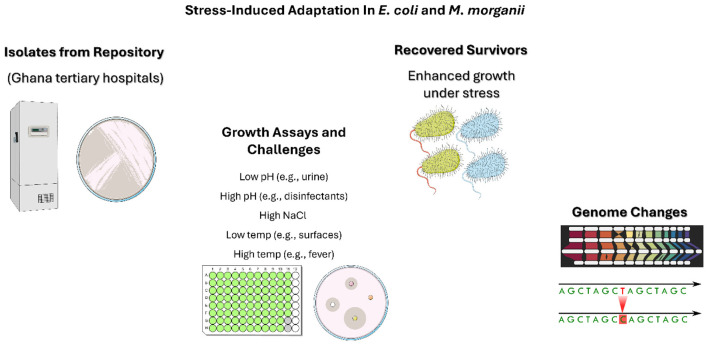
Phenotypic stress-adapted variants and genomic changes in clinical isolates from Ghanaian Tertiary Hospitals.

## Introduction

Bacteria encounter diverse environmental stressors, including nutrient limitation, osmotic pressure, extreme temperatures, acidic conditions, and antimicrobial agents (Poole, 2012). To survive under these harsh conditions, bacteria have protective mechanisms that enable adaptation to both environmental challenges and host-related stressors (Dawan and Ahn, 2022; Jiao et al., 2024). Gram-negative bacteria, including Enterobacterales, employ adaptive mechanisms such as stress response pathways, efflux pumps, and biofilm formation to withstand adverse conditions (Dam et al., 2018). These adaptation strategies contribute to bacterial survival and can enhance antibiotic tolerance and persistence, with potential roles in chronic infections and treatment failures. For instance, stress responses such as the SOS signaling pathway can promote bacterial persistence and biofilm formation, further enhancing survival under hostile conditions (Podlesek and Bertok, 2020). Accurate and sensitive detection of clinically relevant Enterobacterales, including *E. coli* and other opportunistic pathogens, is critical for surveillance and monitoring of tolerant or persistent strains. For example, isothermal transcription amplification using allosteric probes has demonstrated highly sensitive detection of *E. coli* O157:H7 (Long et al., 2024).

Bacterial persistence, a transient, non-heritable state in which a subpopulation of cells survives antibiotic exposure without genetic resistance, is a major challenge in treating infections (Balaban et al., 2019). Unlike resistant bacteria, persisters survive antibiotic treatments due to a dormant or slow-growing state, allowing them to evade immune responses and resume growth later upon withdrawal of antibiotic pressure (Fisher et al., 2017). Various environmental stresses can induce a persister phenotype (Niu et al., 2024). While the molecular mechanisms underlying stress-induced persistence remain poorly understood, this transient adaptation involves modulation of gene expression including metabolism-related genes resulting in phenotypic alterations (Rahman et al., 2025). Changes in gene expression can result from gene amplification, changes resulting from increased mutation rates, and epigenetic inheritance, with additional contributions from the activity of efflux pumps as well as the population structure and heterogeneity of bacterial communities (Motta et al., 2015; Salam et al., 2023).

Bacteria that withstand environmental stresses often possess an array of virulence factors and can rapidly develop novel resistance mechanisms that favor survival. They can also adopt evasion strategies such as swarm motility and biofilm formation (Abban et al., 2025). Biofilms contribute significantly to antibiotic tolerance and chronic infections by protecting bacterial cells from antimicrobial agents and host immune defenses (Hall-Stoodley et al., 2004; Uruén et al., 2020). Recent advances in genome sequencing have revealed that stress-adapted bacteria have acquired genetic mutations that enhance survival in hostile environments (Deatherage and Barrick, 2014). Missense mutations in regulatory genes, *crp* and *hns* result in loss of repression of the *mdtEF* operon, leading to increased production of the MdtEF efflux pump and activation of stress pathways that contribute to increased antibiotic resistance in *E. coli* (Cho and Misra, 2021). However, the contributions of genomic adaptations to antibiotic persistence and bacterial survival remain poorly defined, particularly in *Morganella morganii*, an emerging clinically relevant opportunistic pathogen (Paul et al., 2005; Shi et al., 2022).

This exploratory study investigated how genomic changes resulting from growth stressors influence phenotypic traits and antibiotic response of *E. coli* and *M. morganii* isolates from Ghanaian Tertiary Hospitals. Isolates recovered from growth under stress conditions were treated with an increasing concentration of antibiotics to enumerate persister-like subpopulation cells. Whole genome sequencing of these stress-tolerant cells revealed the accumulation of genomic mutations in *E. coli* and *M. morganii*. By elucidating the interplay between stress adaptation, antibiotic persistence, and genomic factors, this study provides new insights into bacterial survival mechanisms that may contribute to treatment failures and chronic infections.

## Materials and methods

### Bacterial strains and culture conditions

The clinical and environmental strains used in this study were archived bacterial strains obtained from ABISA culture library, West African Centre for Cell Biology of Infectious Pathogens (WACCBIP), University of Ghana. The strains included *E. coli* (EC1, EC3, EC4) and *M. morganii* (m5). The two clinical isolates (EC3, EC4) were isolated from septicemia patients, while the environmental isolates (EC1, m5) are from fomite in the surgical ward of tertiary hospitals in Ghana. The frozen stock of the strains (−80 °C) was revived in Luria Bertani (LB) broth (150 rpm, at 37 °C) and then subcultured on MacConkey agar plate for another 18-24 h. Pure colonies of *E. coli* and *M. morganii* strains were used for all the assays.

### Bacterial growth rate experiment

To establish growth kinetics, optical density was monitored over time at 37 °C with shaking at 150 rpm. Overnight bacterial cultures were inoculated from colony-purified stocks, and this overnight growth was subsequently standardized in 10 ml sterile LB broth in a 15 ml falcon tube to 0.05-0.1 optical density units at 600 nm wavelength, equivalent to 5 x 10^7^-1x10^8^ cells/ml (CLSI, 2020). The OD_600_ was measured at 1 h intervals by transferring 200 μL of culture into a well. This was done in replicates using a microplate reader (Varioskan LUX 1.00.30) to produce growth curves. From the resulting curves, the area under the growth curve (AUC) was calculated from OD_600_ vs. time data using GraphPad Prism (trapezoidal rule, full time interval).

### Antimicrobial susceptibility of strains

The antibiotic susceptibility profiles of the bacterial strains were assessed using the disk diffusion method following Clinical Laboratory Standards Institute (CLSI) guidelines (M100-30^th^ Edition, CLSI, 2020). Briefly, pure colonies were then suspended in Mueller-Hinton broth (MHB) and adjusted to an OD_600_ 0.1, corresponding to 0.5 McFarland standard. The standardized inoculum was spread uniformly across Mueller-Hinton agar (MHA) plates, after which antibiotic-impregnated disks were aseptically placed on the surface (Abiola et al., 2018). The classes of antibiotics tested were fluoroquinolones (ciprofloxacin, 5 μg) and carbapenems (meropenem, 10 μg), which are typically prescribed for *M. morganii* and *E. coli* infections at the study site. The antibiotics concentrations were manually prepared per manufacturers' instructions and 20 μl each were impregnated in sterile filtered disks (6 mm) and allowed to dry before use. Data interpretations were guided by CLSI (2020) breakpoints and categorized as sensitive (S), intermediate (I) or resistant (R). The assays were conducted as two biologically independent replicates.

### Minimum inhibitory concentration determination by broth microdilution method

To assess variations in antibiotic resistance levels among the strains, the broth microdilution method was employed using sterile 96 well microtiter plates. Briefly, a loopful of colony growth was inoculated into 10 ml of MHB and incubated with shaking (150 rpm) at 37 °C for 24 h in a 15 ml Falcon tube (Luber et al., 2003). The test inoculum was prepared by diluting overnight cultures to an OD600 of 0.09–0.1 (~0.5 McFarland; previously confirmed by CFU enumeration). Each well was filled with 100 μl of bacterial culture and 100 μl of 2-fold serial dilutions of antibiotics (ciprofloxacin: 5-1,280 μg/mL diluted from a stock of 20 mg/mL; meropenem: 2.5-640 μg/mL diluted from a stock of 20 mg/mL). Microplates were incubated statically at 37 °C for 24 h, and growth was assessed by OD600 using a microplate reader (Varioskan LUX 1.00.30) at a wavelength of 600 nm. Positive control samples were bacterial cultures without antibiotics, while uninoculated MHB served as the negative control. Experiments were performed in three independent biological replicates. MIC values were reported as the lowest antibiotic concentration showing no detectable growth ( ≤ 5–10% relative to the untreated control).

### Conditional growth assay

To evaluate bacterial adaptability under frequently encountered stress conditions, strains were exposed to a range of physiological stressors, including acidic (pH3) and alkaline (pH 9, 10), temperature (45 °C and 4 °C), and high osmotic pressure. All cultures were grown with shaking at 150 rpm, and optical density was measured in a microtiter plate as described above. Overnight cultures were normalized to an OD600 of 0.1 prior to use. For pH stress experiments, 1 mL of normalized culture was inoculated into 10 mL of LB broth adjusted to the desired pH. Acidic medium (pH 3) was prepared by adding 1.0 N HCl until the final pH reached 3.0. Alkaline media (pH 9 and 10) were prepared by titrating LB with 50% (w/w) NaOH stock in small increments while continuously monitoring the pH using a pH meter, until the final pH reached 9.0 or 10.0 respectively. Cultures in unmodified LB (pH 6.8 ± 0.2) served as the control. Temperature stress was achieved by inoculating 1 ml of prepared culture into 10 ml broth and incubated at either 4 °C (cold stress) or 45 °C (heat stress) for 10 h. Control cultures were incubated at 37 °C, the optimal condition for mesophilic bacteria (Tankeshwar, 2019). Tolerance to severe osmotic stress was imposed using LB supplemented with 10% (w/v) NaCl (~1.71 M), prepared by mixing equal volumes of LB and a 20% NaCl stock prior to inoculation; this high concentration was used as a supra-physiological stressor to induce conserved stress-response pathways. For osmotic stress experiments, 1 mL of pre-cultured bacterial suspension was inoculated into 10 mL of the prepared 10% (w/v) NaCl solution and incubated at 37 °C. The growth rate under different stress conditions was determined at 1 h intervals for 10 h by measuring the OD_600_. The area under the growth curve (AUC) was calculated from OD600 vs. time data using GraphPad Prism (trapezoidal rule, full time interval). After the growth period, bulk cultures were harvested by centrifugation (5,000 × g, 5 min), washed twice with sterile LB, and genomic DNA was extracted for whole-genome sequencing to capture population-level genetic variation. Only stress-exposed populations that reproducibly resumed growth under standard, non-stress conditions were selected for sequencing.

### Antibiotic tolerance and persistence assays

To investigate the impact of growth stressors on subsequent antibiotic responses, including persister cell formation, the stress-recovered variants were subsequently exposed to 2x MIC concentrations of meropenem and ciprofloxacin determined for parent cultures (see above). The growth rates of these strains were compared to the unstressed control cultures. This was achieved as previously described by Kamruzzaman and Iredell (2019) with slight modifications. Briefly, recovered cultures were incubated overnight and were then adjusted to an OD_600_ of 0.3-0.4 units. Antibiotics were prepared at 2x the MIC, including ciprofloxacin (1280 μg/mL for EC1 and EC3 and 640 μg/mL for m5 and EC4) and meropenem (20 μg/ml for all isolates; Supplementary Table 2) were made in MHB. A total of 500 μl of the culture was inoculated into 5 ml of antibiotic containing MHB in a 15 ml falcon tube and incubated at 37 °C with shaking (150 rpm) for up to 8 h. Bacterial cultures were collected at 1 h, 3 h and 5 h of antibiotic exposure, serially diluted and 10 μl of undiluted (10^0^) and diluted samples were plated onto LB agar plates and incubated at 37 °C for 18 h. Colony -forming units (CFU/mL) were calculated and expressed as log_10_ CFU/mL. Samples collected prior to antibiotic exposure (Ini) serve as untreated controls and represent the starting inoculum. The limit of detection was 100 CFU/mL based on the plated volume. In parallel, aliquots (100 μl) were taken at 0, 3 h, 6 h, 8 h and added to a microtiter plate for OD_600_ measurements to assess population-level survival to antibiotics exposure.

### Whole Genome Sequencing (WGS) and variant analysis

Genomic DNA was extracted from parental isolates (EC1, EC3, EC4, and m5) and their corresponding variants derived from exposure to phenotypic stress (see above). Whole-genome sequencing was performed on stress-recovered variants from two *E. coli* isolates (EC1 and EC3) and one *Morganella morganii* isolate, selected as representative strains. Sequencing was performed on bulk recovered populations rather than single-colony isolates to obtain sufficient DNA yield and capture population-level genetic variation. Because sequencing was performed on bulk populations, some variants may represent pre-existing subclones enriched under stress rather than *de novo* mutations. DNA extraction was performed using the QIAmp DNA Mini Kit (QIAGEN), following the manufacturer's protocol. The quality and quantity of extracted genomic DNA (gDNA) were evaluated via electrophoresis through a 1% agarose gel and quantified using the Qubit dsDNA High Sensitivity Assay kit on a Qubit 4.0 fluorometer (Life Technologies). Samples with concentrations ≥200 ng/μl were selected for sequencing. Library preparation and sequencing were conducted at the Next Generation Sequencing laboratory of WACCBIP, University of Ghana. Libraries were prepared using the Illumina DNA Prep kit, following standard manufacturer's protocols. The size distribution and quality of libraries were assessed using the Agilent 4,200 Tapestation system. Final library quantification was performed with the Qubit 4.0 fluorometer. Barcoded libraries were normalized, pooled at equimolar concentrations, diluted to 100 pM, and spiked with 5% Phix Control v3 before sequencing. Paired-end sequencing (2 x 150 bp) was carried out on the Illumina iSeq100 platform using the iSeq 100 i1 Reagent v2 (300-cycle).

### Data processing and quality assessment

Raw sequencing reads (FastQ format) were assessed for quality using FastQC and summarized using MultiQC (Ewels et al., 2016). Trimming of low-quality reads was performed using Trimmomatic, retaining reads with Phred score >Q32. Genome assemblies were generated using SPAdes v3.13.1 and assembly quality was evaluated with CheckM (v1.2.3), which assesses genome completeness and contamination based on lineage-specific marker genes (Parks et al., 2015). Genomes with >5% contamination and < 90% completeness were excluded from further analysis.

### Bioinformatics pipeline for variant calling

To identify genomic variants, raw Illumina sequencing reads were aligned to their respective reference genomes: *E. coli* K-12 (NC_000913.3) and *M. morganii* (NZ_CP034944.1), respectively. Variant calling was performed using Snippy v4.6.0 (https://github.com/tseemann/snippy), a pipeline that detects high-confidence genomic differences including single nucleotide polymorphisms (SNPs) and insertions/deletions (indels) between the sequencing reads and a reference genome. Snippy employs sub-routines BWA-MEM for read alignment, FreeBayes for variant calling via Bayesian statistics models, and Snpeff for functional annotation of identified variants. Variants were called at positions with a minimum read coverage of 10x, and a minimum variant allele fraction of 90 %, and reads with base quality < 13 or that did not map to the reference were ignore, minimizing the impact of potential low-level contamination. Snippy detects base substitutions such as SNPs, multiple nucleotide polymorphisms (MNPs), complex variants (combination of SNPs and MNPs), and indels. Variants were categorized by SnpEff as either synonymous or missense variants based on their predicted protein effects. Stress-associated variants were defined as missense variants present in stress-recovered populations but absent in the corresponding parental strains. These variants were extracted using R v4.4.0 and visualized using UpSet plots, and bar charts.

### Statistical analysis

All analyses were descriptive. The experiments were conducted in two independent biological replicates, unless otherwise noted, to ensure reproducibility. The bacterial growth under different stress conditions was summarized by calculating the area under the growth curve (AUC). Data are presented as the means of two independent experiments and error bars indicate the standard error of the mean (SEM) for descriptive visualization of replicate variability. Due to the exploratory nature of the study, no inferential statistical tests were performed due to a low number of biological replicates.

## Results

### Impact of stress on *E. coli* and *M. morganii* growth kinetics

**Figure 1:**
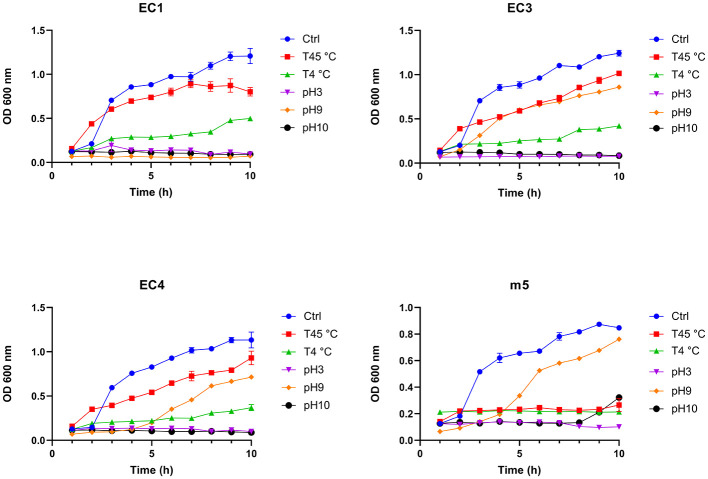
Growth curves of *E. coli* (EC1, EC3, EC4) and *M. morganii* (m5) strains at OD 600nm under various stress conditions: temperature at 45 °C (T45 °C) and 4 °C (T4 °C), and varying pH conditions (pH 3, pH 9, and pH 10). Control (Ctrl) represents optimal growth conditions (37 °C, pH 6.8). Data are presented as mean ± SEM from two independent biological replicates (*n* = 2). Due to the limited number of biological replicates, no formal statistical testing was performed, and no significance comparisons are reported. Area under the curve (AUC) was used as a descriptive measure to summarize overall growth trends across conditions (Supplementary Table 1). While the limited power of replicates precludes formal statistical analysis, we note that the duplicate measurements are in close agreement.

The effects of the phenotypic stressors on bacterial survival were assessed for *E. coli* (EC1, EC3, EC4) and *M. morganii* (m5) isolates by measuring the optical density (OD_600_) for 10 h, as evaluated relative to standard optimal growth conditions for mesophilic bacteria (37 °C, pH 6.8 (Figure 1).

The AUC analysis of OD600 growth curves revealed strong, condition-specific and strain-dependent effects of environmental stress on population-level growth and tolerance dynamics (Supplementary Table 1). When grown at the optimum conditions, OD_600_ values of 1.0-1.4 units are achieved; while *E. coli* isolates have a lag phase of 1-2 h and are reaching stationary phase at 10 h, *M. morganii* appears to initiate shallow exponential growth immediately and is not yet in stationary phase at 10 h. At 37 °C (control), all EC strains showed high AUC values (7.1–7.7), while m5 had a lower value of 5.6. Growth decreased at 45 °C, with AUC values ranging from 5.2 to 6.4 in the EC strains and 2.0 in m5. Growth at 4 °C was low for all EC strains (2.0–2.8), and no measurable value was obtained for m5. At pH 3, growth was strongly reduced in all strains (0.67–1.20). At pH 9, EC3, EC4, and m5 maintained moderate growth (3.0–5.0), whereas EC1 grew poorly (0.56). EC3 tolerated alkaline stress best (pH 9 AUC = 4.96). At pH 10, all strains showed very limited growth (AUC < 1.4). Under osmotic stress, exposure to high NaCl (1.71M) resulted in a reduction in OD600 across all strains relative to the control condition (Supplementary Figure 1). Overall, the data show that 45 °C and pH 9 allowed some level of tolerance, while acidic and highly alkaline conditions restricted survival across strains. m5 generally had lower AUCs than EC strains but still maintained moderate survival at pH 9. Most of the strains thrive at 45 °C and pH 9 compared to other growth stressors.

### Impact of prior temperature exposure on growth kinetics

**Figure 2:**
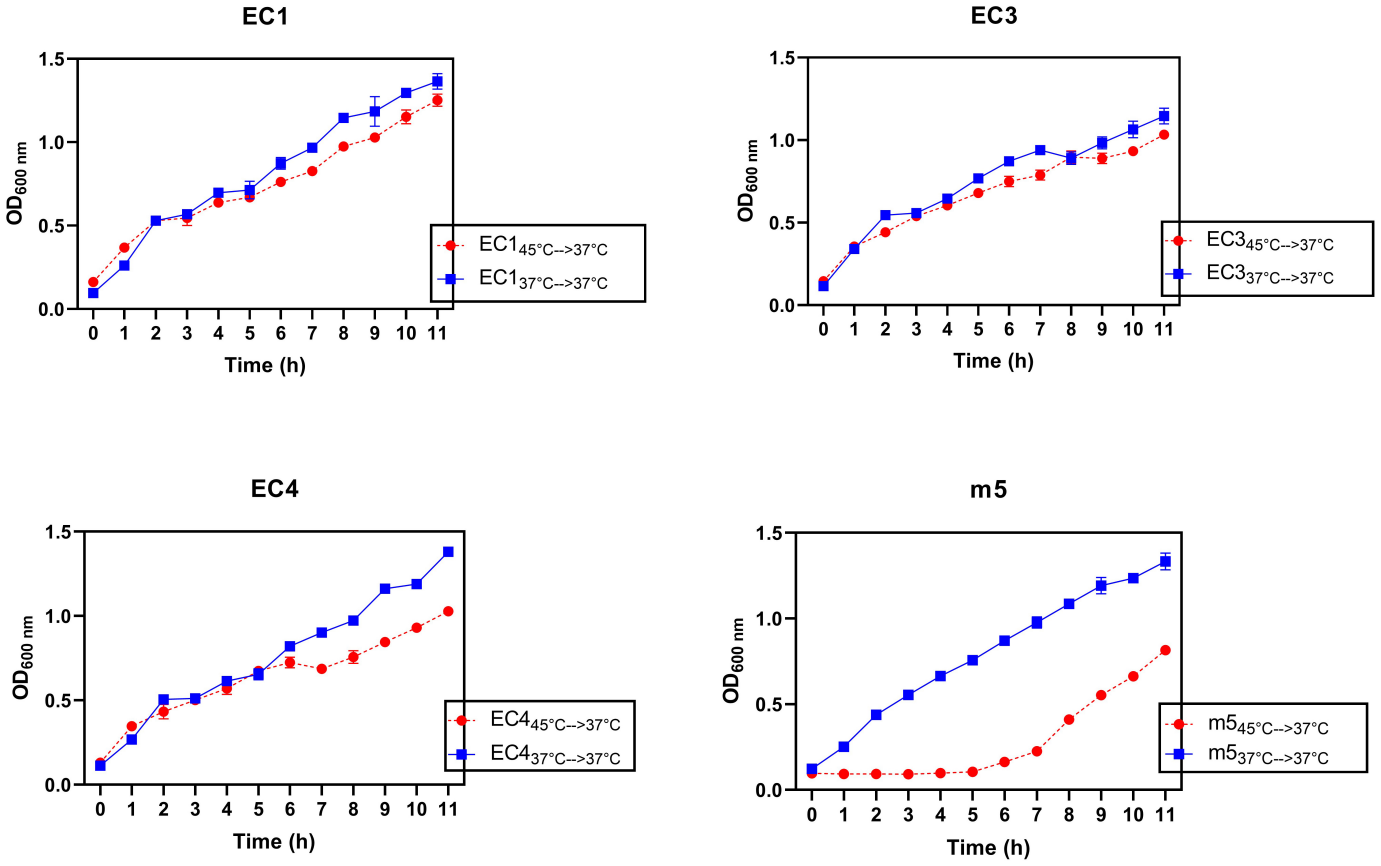
Effect of thermal tolerance on growth of isolates. Temperature-tolerant variants were recovered in fresh medium and grown at 37 °C after prior exposure to 45 °C (45 °C–>37 °C) and compared to unstressed controls grown continuously at 37 °C (37 °C–>37 °C). Error bars represent the standard error of the mean (SEM) from two independent biological replicates (*n* = 2). While the limited power of replicates precludes formal statistical analysis, we note that the duplicate measurements are in close agreement.

To assess the physiological impact of prior heat exposure (45 °C), temperature-tolerant variants were recovered in fresh medium, and their growth kinetics were compared to unstressed controls previously grown at 37 °C (Figure 2). While EC isolates show substantial regrowth after heat exposure, the m5 isolate is dramatically impacted. The temperature-tolerant variant of EC1 reached an exponential growth phase around same time as the control (2h) and with approximately equivalent OD_600_ values at 11 h (1.4 and ≈1.45). In EC3, the heat-stressed variant showed a lag in growth of ~1h in entering exponential phase and consistently lowered OD values throughout the growth period. The recovered variant (EC3_temp_) reached an OD600 of 1.25 in 11 h, compared to the control of ~1.42, resulting in a reduction of ~12% in stationary phase density. In EC4, the heat-stressed variant reached OD_600_ ≈ 1.05 at 11 h relative to the control (OD_600_ ≈ 1.43). While there may be subtle differences between the heat recovered and the control cultures, overall, they largely follow similar growth pattern.

The most pronounced impact was observed for the *M. morganii* isolate (m5), with the heat-stressed variant showing a 5 h lag before any detectable growth, whereas the control sample began growing by the 1 h measurement. At 11 h, the heat-stressed variant was at OD_600_ ≈ 0.8, compared to OD_600_ ≈ 1.45 in the control, a 45% reduction in the final OD_600_, indicating stress sensitivity and delayed bacterial recovery.

### Effect of growth stressors on antibiotic tolerance

To quantitatively assess phenotypic changes induced by growth stress on the EC1, EC3, and m5 isolates, their susceptibility to meropenem and ciprofloxacin was determined followed by an assay to measure tolerance. All isolates were exposed to stress conditions (acidic and alkaline pH, heat, and osmotic stress) and recovered stress populations were challenged with meropenem and ciprofloxacin. All tested isolates including the control samples not exposed to stress exhibited resistance based on clinical definitions, with MICs of ≥10 μg/mL for meropenem and ≥320–640 μg/mL for ciprofloxacin (Supplementary Table 2).

**Figure 3:**
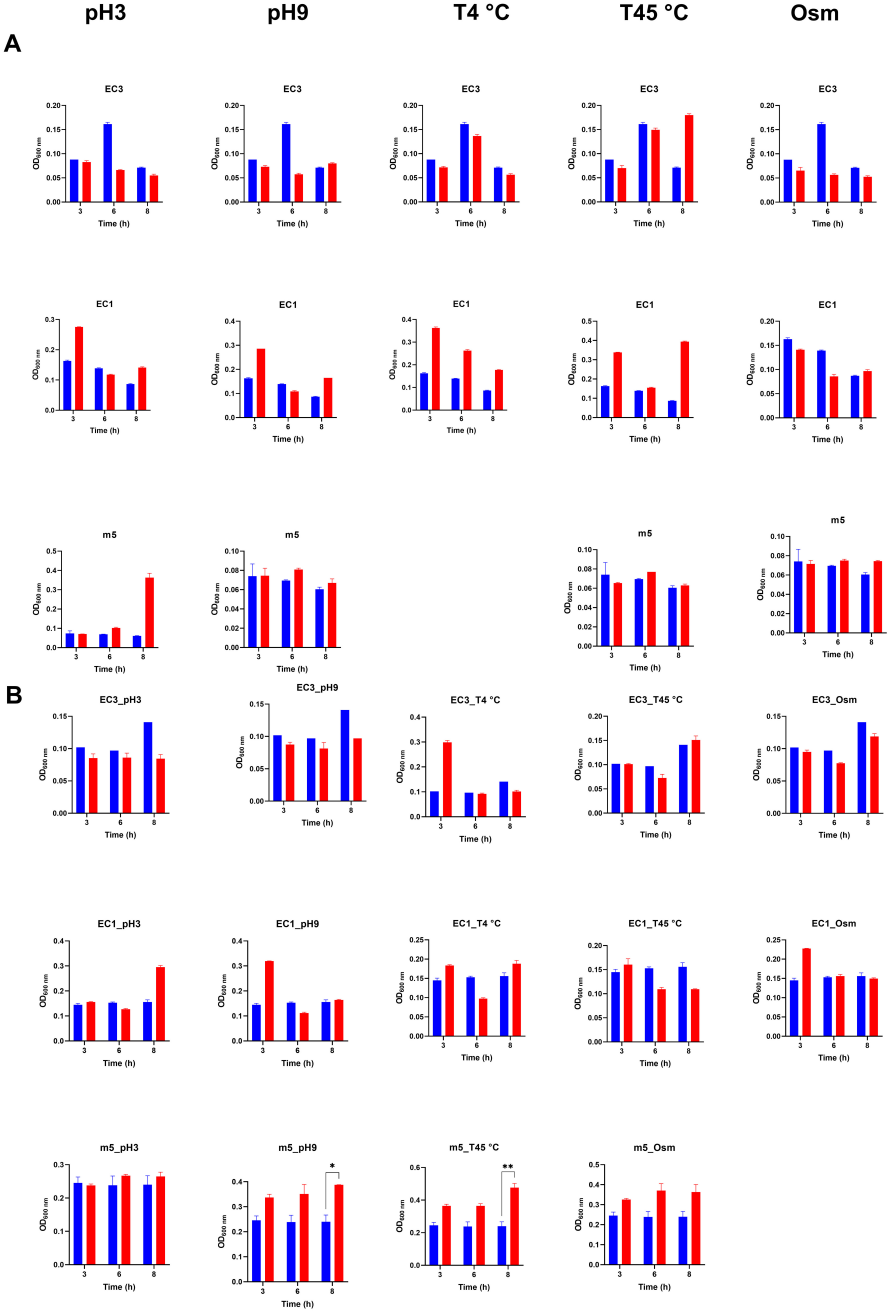
Optical density (OD600) measurements of stress-recovered *E. coli* strains (EC1, EC3) and *Morganella morganii* (m5) upon two consecutive antibiotic treatments (n = 2 biological replicates) and two technical replicates. **(A)** Meropenem treatment and **(B)** Ciprofloxacin treatment. Stressed variants are shown in red; unstressed controls are shown in blue. Error bars represent the standard error of the mean (SEM). While the limited power of replicates precludes formal statistical analysis, we note that the duplicate measurements are in close agreement.

Isolates were challenged with 2x MIC concentrations of ciprofloxacin and meropenem for the parental strains, and the OD_600_ was measured at 3 points (3, 6 and 8 h). Exposure of *E. coli* strains (EC1, EC3, EC4) and *M. morganii* (m5) to diverse environmental stresses (acidic and alkaline pH, heat, and osmotic stress) prior to testing against meropenem (Figure 3a) and ciprofloxacin (Figure 3b) altered population-level growth dynamics. Across strains, cells exposed to acidic pH, alkaline pH and osmotic stress maintained measurable growth despite the antibiotic challenge. In contrast, heat-stressed populations (45 °C) displayed variable effects, with some conditions showing no difference compared to unstressed controls.

### Quantification of Meropenem and Ciprofloxacin Persister-like subpopulation of cells

**Figure 4:**
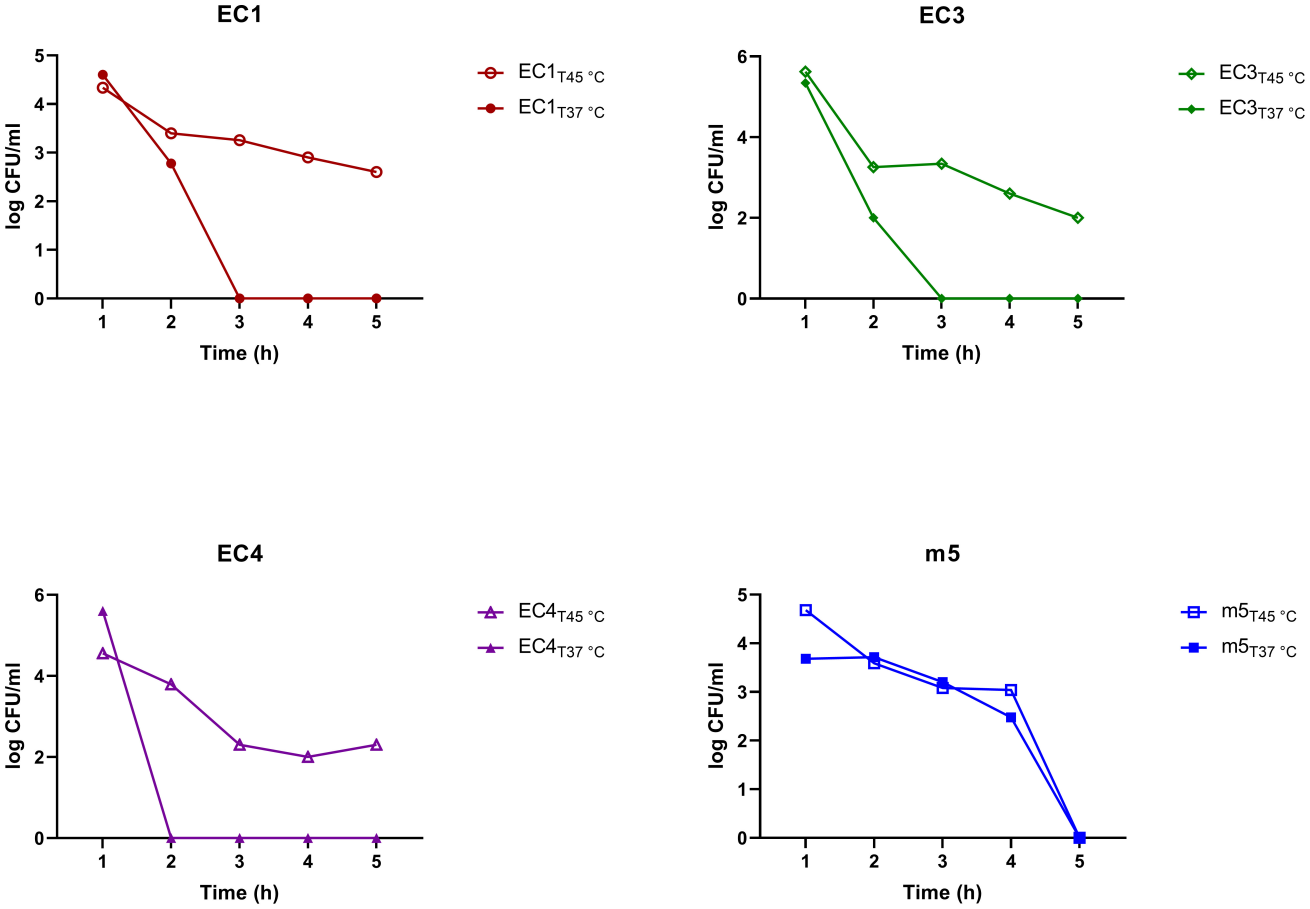
Differences in persister-forming ability of *E. coli* (EC1, EC3, EC4) and *Morganella morganii* (m5) strains. Strains were exposed to two times the minimum inhibitory concentration (2 × MIC) of meropenem or ciprofloxacin. Growth and survival were monitored over 5 h using optical density (OD600) measurements and colony-forming units (CFU/ml). Assays were performed with two biologically independent replicates (*n* = 2), and error bars represent the standard error of the mean (SEM); while the limited power of replicates precludes formal statistical analysis, we note that the duplicate measurements are in close agreement. Observed subpopulations are consistent with persister-like behavior; however, true persisters were not definitively confirmed. Statistical analyses were not performed due to exploratory nature of the study.

Time-kill assays measuring colony-forming units (CFU/ml) and optical density (OD_600_) were measured over five hours to evaluate biphasic growth of the isolates upon treatment with meropenem and ciprofloxacin at 2 × MIC values (Figure 4). For *Meropenem*, a rapid initial decline in CFU/ml (~ a 5-log reduction relative to the starting inoculum) was observed after 1 h, consistent with a biphasic killing pattern that is characterized by an early bactericidal effect followed by the survival of a subpopulation of cells. At the 1-h mark, most strains exhibited a sharp decrease in CFU/ml, followed by a gradual decline over the next 4 h. OD_600_ values remain low and stable (~0.08–0.09), indicating that no significant cell lysis or regrowth occurred during the remaining hours during meropenem treatment. For Ciprofloxacin, OD_600_ readings showed a gradual increase after the initial drop for some strains, but remained below 0.2, indicating limited growth recovery. CFU counts exhibited variability among strains, with some showing transient increases before declining suggesting heterogeneous persister-like behavior.

### Effect of temperature stress on meropenem persister-like subpopulation of cells

**Figure 5:**
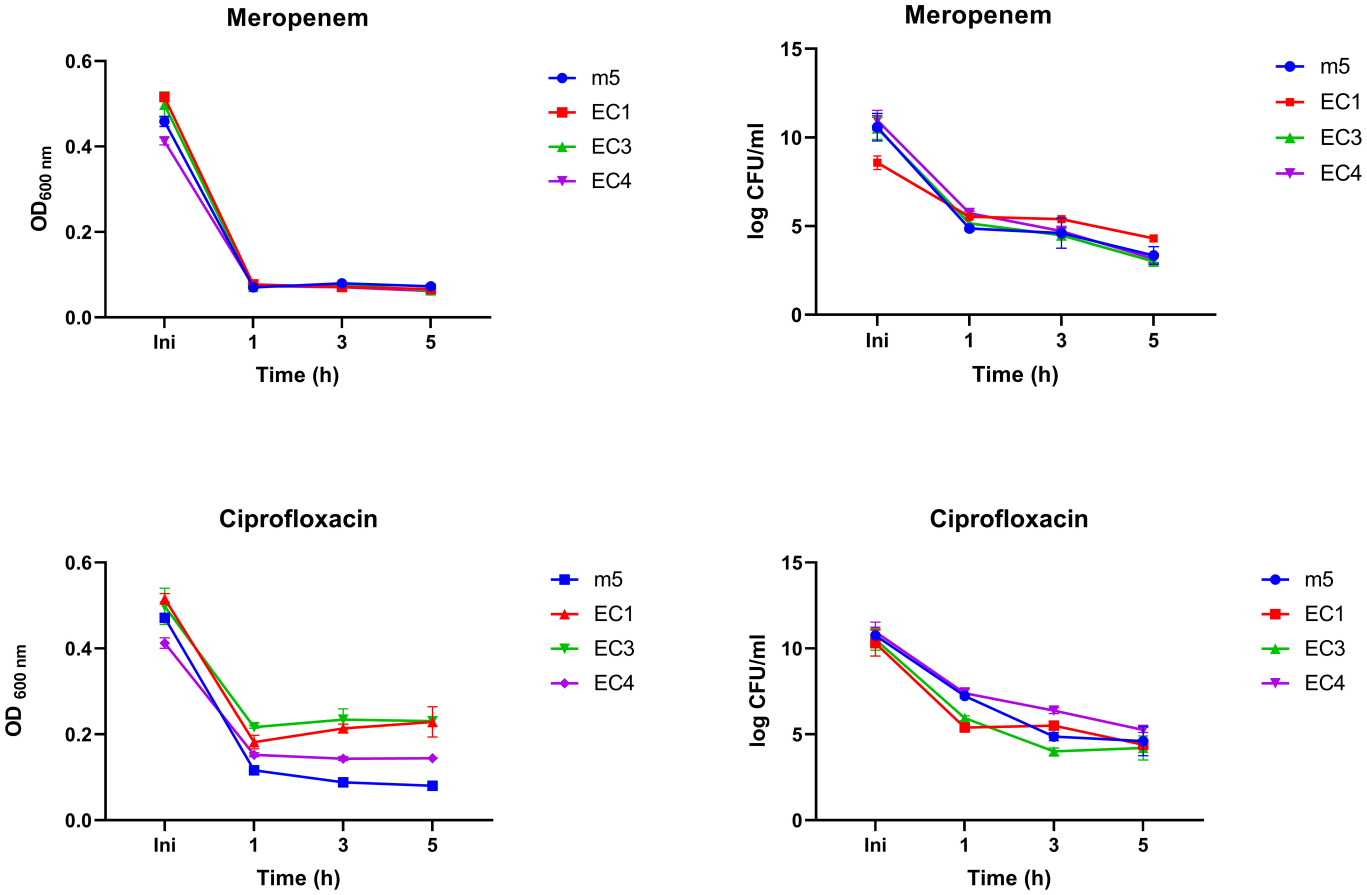
Differences in persister-forming ability of temperature-stress recovered *E. coli* strains. (EC1T45 °C, EC3T45 °C, EC4T45 °C) and *M. morganii* (m5T45 °C) under meropenem exposure. Cells were recovered from 45 °C stress (T45 °C) and compared to unstressed controls (T37 °C). Survival over time was assessed using a time-kill assay, measured as colony-forming units (CFU/ml).

It was further investigated whether prior exposure to heat stress (recovered after growth at 45 °C) enhanced the persister levels of the strains to meropenem. The unstressed control strains of *E. coli* similar to the above (Figure 4), exhibited a steep decline in CFU/ml of approximately 5-log values within 2-3 h of meropenem exposure (Figure 5). In contrast, temperature-recovered EC variants displayed prolonged survival rates, losing only 2-3 log values over 5 h. Among the heat-stressed variants, the EC1_T_45 °C maintained higher CFU/ml counts compared to their unstressed counterparts. EC1_T_45 °C and EC3_T_45 °C retained viable populations even after 4-5 h of exposure. EC4 and m5 temperature-recovered variants exhibited a more gradual decline. Notably, the m5_T_45 °C and control samples maintained essentially the same CFU/ml until hour 5, when both dropped to zero.

### Genomic signatures of *E. coli* and *M. morganii* cultures after phenotypic stress adaptation

To establish the genomic background of the experimental isolates, whole-genome sequences of the parental *E. coli* (EC1, EC3, EC4) and *M. morganii* (m5) strains were compared with their respective reference genomes (*E. coli* K-12 MG1655 and *M. morganii* ATCC 25830). Genome mapping metrics are summarized in Supplementary Table 4, and the corresponding variant list is provided in Supplementary Data Sheet 1. Variant calling against *E. coli* K-12 identified **2,225, 2,166**, and **2,217** missense variants in EC1, EC3, and EC4, respectively, indicating substantial genomic divergence from reference strain. The overall gene content was similar except for a few additional hypothetical proteins in the studied isolates. Functionally, the mutations were enriched in genes associated with antimicrobial resistance, including chromosomal loci previously linked to fluoroquinolone resistance (*gyrA, parC, parE*), as well as components of multidrug efflux pumps (*acrA, acrB, marA, tolC*). Several virulence-associated genes also carried nonsynonymous substitutions, such as *fimH, ompW, fhuF, umuC, umuD, kdp, flgM, mgtA*, and *hdeA*. Comparison of the *M. morganii* parental strain m5 to the reference ATCC 25830 genome identified variants in 893 genes. Several were in antimicrobial resistance determinants, including *cat, gyrA, parC, parE, acrA, tolC, mgrB, mdtA*, and *mdtC*. Mutations were also observed in virulence-associated genes involved in motility, chemotaxis, and environmental adaptation, such as *fumA, motB, mgtC, flg, fliF, tap, fimD, phoP, phoQ, phoC*, and *rsm* family regulators.

### Missense mutations in the stress-exposed *E. coli* isolates

**Figure 6:**
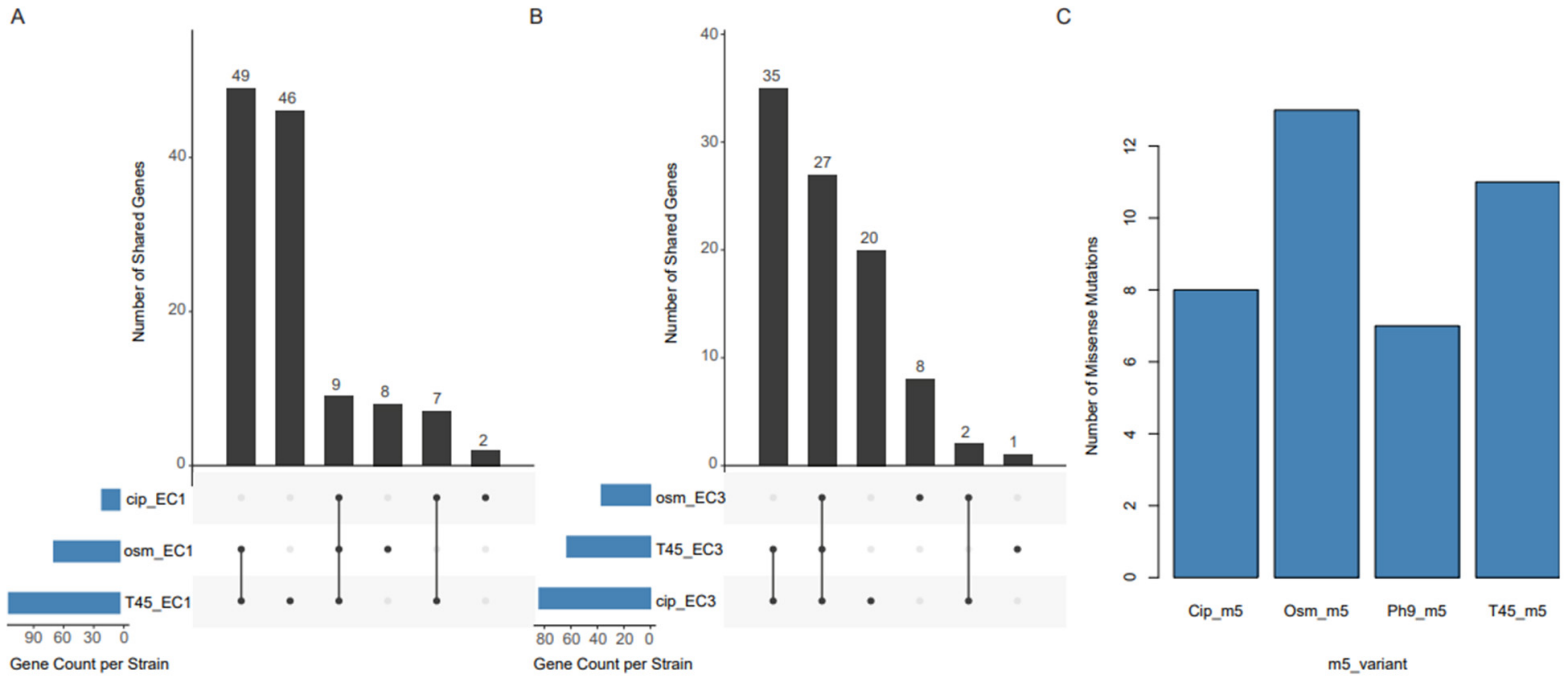
Comparison of the number of unique variants detected in *E. coli* and *M. morganii* stressed variants. (cip, ciprofloxacin recovered; osm, osmotic stress recovered; ph9, alkaline stress recovered; T45, temperature at 45 °C recovered) relative to the unstressed controls/parental strains. **(A)** Upset plot showing intersection of the unique variants among shared genes across conditions in EC1. **(B)** Upset plot showing the intersection of the unique variants among shared genes across conditions in EC3. **(C)** Bar plot showing the number of variants in the m5 stress-recovered strains relative to unstressed control parental culture.

To identify genetic changes, including the potential for enrichment of pre-existing changes, associated with stress adaptation, unique missense variants present exclusively in the sequenced bulk culture DNA of stressed populations were considered as signatures arising from each stress. Stressed populations recovered at temperature 45 °C, pH 9, osmotic and ciprofloxacin stress from EC1, EC3, and m5 were sequenced. Alkaline pH from *E. coli* strains have above 10 % contamination based on checkM analysis and were thus removed from the analysis. Comparative analysis of stressed *E. coli* populations with their respective parental genomes revealed condition-specific missense variants associated with temperature, osmotic, and ciprofloxacin stresses (Figure 6). The number of unique variants detected varied across treatments, with temperature-tolerant (T45_EC1) harboring unique mutations in 41 genes, the osmotic-stress variant (osm_EC1) had 8, and the ciprofloxacin-exposed variant (cip_EC1) carried 47. A total of 48 gene changes were shared across temperature and osmotic-recovered variants in EC1, 11 between T45_EC1 and cip_EC1, and all three variants shared 12 genes changes (Figure 6A). In EC3, ciprofloxacin exposure (cip_EC3) produced 20 unique missense variants, osmotic stress (osm_EC3) led to 8, and elevated temperature (T45_EC3) to 35 (Figure 6B). In the *E. coli* strains, temperature adaptation was associated with mutations in *rrrD, arsB, yjjV, lacZ, flgE, ftsK*, and transposase genes belonging to the *ISNCY* and *IS4* families. Ciprofloxacin-stressed cells displayed mutations in *potB, ggt, fldL, draB, cbeA, puuE, rrrD, baeS*, and *ompC*, in addition to *IS4* family transposase genes. Some of these genes (*baeS, ompC, potB*) are linked to envelope stress response, efflux regulation, and membrane integrity, suggesting that fluoroquinolone exposure may select for variants enhancing multidrug tolerance and cellular adaptation. There are also genes with poorly characterized functions, including *yehF*, which is regulated by *lexA*, a known stress response gene. The *scpC* gene has been reported as an essential virulence factor in *Streptococcus* spp.; however, there is little or no information on the function of *yieH, yihR*, and *yjgL*.

### Missense mutations in the stress-exposed *M. morganii* cultures

To investigate genomic signatures associated with stress adaptation, variants unique to each stressed *M. morganii* variants were compared to those in the parental strain, m5 (Figure 6C). The ciprofloxacin-exposed variant (cip_m5) harbored eight unique missense variants, including single-nucleotide polymorphisms (SNPs) and multinucleotide polymorphisms (MNPs). Most of these mutations occurred in genes encoding hypothetical proteins, while one was located in *cysJ*, which encodes the flavoprotein subunit of sulfite reductase. Alterations in *cysJ* may influence sulfur metabolism and oxidative stress response, potentially contributing to ciprofloxacin tolerance. Exposure to osmotic stress resulted in 13 unique missense variants distributed across *cysJ*, several hypothetical proteins, and an *IS3*-family transposase. The alkaline stressed isolate (ph9_m5) exhibited seven unique missense variants, mainly affecting transposase-encoding genes and hypothetical proteins. The recurrent appearance of transposase mutations under different stress conditions implies a potential role of mobile genetic elements in the stress response and genome plasticity of *M. morganii*. The temperature-tolerant variant (T45_m5) possessed 11 distinct mutations in *rrrD-6, IS3*-family transposase, and several hypothetical proteins. The *rrrD* locus is a prophage-related region, and mutations there could reflect stress-induced prophage activation or recombination events.

## Discussion

Antibiotic-resistant pathogenic bacteria are a major driver of infectious diseases, which remain one of the leading causes of human mortality worldwide (Mancuso et al., 2021). The World Health Organization (WHO) has identified and categorized many of these bacteria as priority pathogens. Among the most critical of these are carbapenem-resistant Enterobacterales, including *E. coli* and *M. morganii*, which pose significant treatment challenges in clinical settings (Zhuang et al., 2025). Clinical studies have shown that bacterial resistance and persistence traits can significantly influence treatment success and patient outcomes in infections with carbapenem-resistant Gram-negative bacteria (Zhuang et al., 2025, 2023). These findings underscore the potential impact of stress-induced tolerance on therapeutic efficacy in hospital settings. The *M. morganii* and *E. coli* isolates studied here were obtained from cases of septicemia and environmental fomites within the intensive care units of Ghana tertiary hospitals, highlighting their potential roles in healthcare-associated infections. The ability of bacterial pathogens to survive and adapt under diverse environmental stressors is essential to their antibiotic persistence in clinical and ecological niches.

This exploratory proof-of-concept studyevaluated the growth responses of *E. coli* (EC1, EC3, and EC4) and *Morganella morganii* (m5) isolates to a range of frequently encountered stressors including a growth temperature above optimum, acidic and alkaline pH values, and hyperosmotic condition. Subsequently, persister-like cells are generated, as evaluated under selection by ciprofloxacin and meropenem antibiotics. The applied stress conditions represent extreme but biologically informative proxies rather than exact replicas of host niches. Supra-physiological temperature, pH, and osmotic stress were used to robustly induce conserved stress-response pathways known to contribute to antibiotic tolerance and persister formation. While these conditions exceed those typically encountered *in vivo*, bacteria may experience transient or localized exposure to similar stresses in host-associated, hospital or environmental settings. This approach enables systematic assessment of stress-induced phenotypic heterogeneity. Collectively, our findings highlight a complex interplay between species-specific stress tolerance, growth adaptation, and persister-like phenotypes with key implications for antimicrobial resistance and survival under hostile conditions. These results provide insight into stress resilience and potential survival strategies among Enterobacterales. The observed variation in survival across temperature and pH conditions highlights the distinct stress tolerances of *E. coli* and *M. morganii* isolates in this study. The four isolates exhibited reduced growth at 45 °C, with OD values suggesting moderate thermal tolerance of *E. coli* isolates and minimal thermal tolerance of the *M. morganii* isolate. Temperature is reported to be one of the most critical environmental factors influencing the structure and function of biomolecules, especially on the outer membrane of Gram-negative cells (Dash et al., 2022). Exposure to elevated temperature was used as a supra-physiological stress to robustly activate heat-shock and proteotoxic stress responses. Exposure to high NaCl resulted in a consistent reduction in OD600-based biomass accumulation across all strains relative to the control condition, with greater tolerance in *M. morganii*, m5. The osmotic stress condition exceeds osmolarities typically encountered in host environments such as plasma or urine, but reflects the impact of severe hyperosmotic stress, which limits normal growth and challenges cellular homeostasis rather than supports physiological adaptations. These results highlight differences in stress tolerance among strains rather than baseline growth. When the growth media was adjusted to other pH values, the stress elicited a more severe inhibition pattern with extreme acidic (pH 3) and alkaline (pH 10) conditions causing complete growth inhibition for the three *E. coli* isolates, reflecting their limited ability to maintain pH homeostasis under extreme stress. Although most human body sites maintain narrower pH ranges, bacteria may experience transient exposure to low pH in the stomach or other acidic skin environments, as well as alkaline conditions in certain niches such as infected wounds, cleaning agents or hospital environments. The growth of *E. coli, Bacillus cereus*, and *Pseudomonas aeruginosa* is affected by changes in pH (Razmi et al., 2023). Survival at pH 4.50 has been reported in some pathotypes of *E. coli* (Vivijs et al., 2016). In this study, *M. morganii* exhibited greater tolerance to alkalinity at pH 9, with modest growth at longer time points, which may reflect a natural adaptation to more variable environmental niches. This is consistent with the biology of *M. morganii* as a urease-positive uropathogen, where urease activity catalyzes the hydrolysis of urea into ammonia and carbon dioxide, thereby increasing local pH (Minnullina et al., 2019). *M. morganii* is commonly associated with urinary tract infections, where alkaline conditions may be encountered (Liu et al., 2016; Sium et al., 2025). The clinical *E. coli* strains (EC3, EC4) show moderate survival at pH 9, compared to the environmental isolate (EC1), suggesting host-adaptive features such as enhanced acid resistance systems or stress-responsive regulators. The enhanced survival of strains under alkaline pH suggests the presence of mechanisms conferring partial tolerance to sublethal alkaline stress. These mechanisms may include differential expression of stress response systems such as the gad operon, alkaline phosphatase regulators, proton efflux systems, and stress-inducible transcriptional regulators such as those activated by envelope-stress pathways (Maurer et al., 2005; Padan et al., 2005). The observed strain-specific responses in the strains investigated here underscore intra-species variability and highlight the potential for stress-driven selection of subpopulations with enhanced fitness. *M. morganii* exhibited a distinct profile, with greater sensitivity to elevated temperature, possibly due to differences in membrane lipid composition or stress response regulatory circuits. These results highlight the importance of environmental stress in shaping bacterial physiology and suggest that adaptive stress responses are not only stress-specific but also strain-and species-dependent. This data also suggests that while acidic pH causes rapid and severe inhibition, thermal stress may allow physiological adaptation or selection of advantageous phenotypic variants. These physiological observations lay a foundation for subsequent molecular investigations including regulation of stress response genes and persistence determinants under similar conditions.

To further understand the consequences of stress adaptation, the growth kinetics of heat-tolerant variants recovered from 45 °C exposure were evaluated in comparison to their unstressed controls. The ability of bacterial strains to survive and recover from thermal stress has direct clinical relevance, particularly in the context of host-associated environments and hospital surface contamination (Cebrián et al., 2017; Liang et al., 2023; Wißmann et al., 2021). Human body temperatures are not uniform, while febrile temperatures in the human host rarely exceed 39-41 °C (El-Radhi, 2012), localized or transient thermal stress may occur during inflammation or abscesses may expose bacteria to even higher, sublethal heat levels above that of surrounding skin (Fierheller and Sibbald, 2010; Rabkin and Hunt, 1960). Among the clinical isolates, EC3 recovered with minimal delay, suggesting a potential for high thermal resilience in patient-derived strains, which may help survival during febrile episodes or in organs with elevated local temperatures. EC4, by contrast showed a more pronounced growth delay, despite its clinical origin, emphasizing that thermal adaptation may be strain-dependent and may not be determined by source. These findings from our exploratory study, although limited by sample size, are particularly relevant in understanding how pathogens withstand immune-mediated fever and how they may persist in healthcare environments where sublethal heat exposure is common. Also, thermal recovery capacity may influence both infection potential and environmental persistence especially in setting where bacteria encounter fluctuating temperatures on the skin, mucosa, invasive devices or inadequately sterilized instruments.

The ability of bacteria to survive high concentrations of antibiotics without undergoing genetic resistance is a hallmark of persister-like subpopulation cells or viable but non-culturable formation, a clinically significant phenomenon linked to chronic and recurrent infections (Fauvart et al., 2011; Zhou et al., 2023). In this exploratory study, all strains exhibited a biphasic killing pattern upon exposure to meropenem, consistent with the emergence of a subpopulation of tolerant cells. The low OD values observed throughout the time kill assay indicate that surviving cells remained in a non-growing, metabolically inactive state that did not experience lysis as would be expected upon exposure to meropenem. This non-heritable tolerance phenomenon has also been documented in various clinically relevant species, including *Acinetobacter baumannii* (Hussain et al., 2025), *Pseudomonas aeruginosa* (Hazan et al., 2014), and *Klebsiella pneumoniae* (Lee et al., 2019) upon meropenem exposure. Importantly, the findings in this study suggest that prior exposure to sublethal heat stress (45 °C) enhances meropenem persister-like formation in the studied *E. coli* and *M. morganii* isolates. The temperature-recovered variants, especially EC1_T_45 °C and m5_T_45 °C demonstrated prolonged survival under meropenem treatment, highlighting that stress preconditioning may induce a physiological state more prone to antibiotic persistence or a viable but non-culturable state. This has also been shown in *Streptococcus mutans*, when preheated at 50 °C produced up to 100-fold more persisters on subsequent antibiotic exposure (Leung and Lévesque, 2012) and trehalose-deficient *E. coli* that showed increased oxidative stress and indole signaling after heat shock resulted to elevated persistence (Kuczyńska-Wiśnik et al., 2015). Substantial variability in persister frequencies in natural isolates of *E. coli* under identical antibiotic treatments has also been reported (Stewart and Rozen, 2012). Likewise, different *E. coli* O157:H7 strains also show markedly different persister levels under controlled conditions (Carter et al., 2024). From a clinical perspective, these results raise concern about the resilience of multidrug-resistant strains under combined stress, such as a febrile host environment followed by antibiotic treatment. Host factors, including microbiota and immune status, may further influence bacterial antibiotic persistence and treatment outcomes. For example, modulation of intestinal barrier function and immunity has been shown to impact microbial resilience and susceptibility to infection (Su et al., 2023). Collectively, these findings reveal that environmental stress adaptation not only shapes growth dynamics but also influences the emergence of antibiotic-tolerant subpopulations. As such, understanding this dynamic is crucial for infection control strategies and developing therapeutics targeting tolerant populations.

Environmental stress is a key driver of genomic adaptation in bacteria, shaping their survival strategies under hostile conditions (Jiao et al., 2024; Rodríguez-Verdugo et al., 2016). In this study, both *Escherichia coli* and *M. morganii*, exposed to heat stress select for subpopulations with enhanced antibiotic tolerance or persistence. These selective pressures can also lead to genetic changes that can confer adaptive advantages (Swings et al., 2017). Comparison of the parental isolates against their respective reference genomes revealed substantial genomic divergence in both *M. morganii and E. coli*, reflecting their clinical origins and evolutionary distance from laboratory reference strains. In *E. coli*, the three parental isolates (EC1, EC3, EC4) exhibited thousands of missense variants relative to K-12 MG1655, with extensive overlap among strains and genes linked to virulence, antimicrobial resistance, and toxin-antitoxin systems. Mutations in fluoroquinolone targets (*gyrA, parC, gyrB, and parE*) and meropenem penicillin binding proteins (PBPs) were particularly notable because they were consistent with observed resistance phenotypes. Similarly, *M. morganii* m5 displayed widespread polymorphisms compared to the ATCC 25830 reference, particularly within resistance (*gyrA, parC, acrA, mdtA, mgrB*), virulence (*motB, phoP/phoQ, fliF*), and TA (*dinJ, pasI*) loci, as well as transposase-related regions. Comparative genomic analyses have shown that clinical *M. morganii* isolates harbor a broader repertoire of antimicrobial resistance and virulence-associated genes compared to the reference strain *M. morganii* (Behera et al., 2023; Sium et al., 2025). Notably, the reference *M. morganii* KT strain has been reported to possess genes associated with virulence and stress adaptation (Chen et al., 2012), supporting the notion that clinical isolates harbor pre-existing reservoirs of adaptive and resistance-associated mutations. These baseline differences emphasize that both species already possess reservoirs of adaptive and resistance-associated mutations that are likely to influence their stress responses.

When exposed to environmental and antibiotic stressors, both studied *M. morganii* and *E. coli* exhibited further genomic plasticity that may be *de novo* mutations due to stress or pre-existing but become enriched following exposure to stressors. Whole-genome sequencing was limited to a subset of isolates, and therefore genomic findings may not capture all stress-associated variation present across the full strain panel. Also, it is important to note that, without a non-stressed passage control, some mutations observed in stress-recovered populations may reflect pre-existing subclonal variation or adaptation to growth in the laboratory rather than being strictly stress-induced. In *M. morganii*, stress adaptation involves missense variants mostly within hypothetical proteins, *cysJ, rrrD*, and IS3-family transposases, suggesting a response mediated through transposable elements and prophage-associated loci. In contrast, *E. coli* isolates accumulated a broader range of mutations under stress, spanning membrane porins (*ompA, ompC*), iron transport and metabolic genes (*fhuA, fepC, entS, serC*), and regulators (*baeS, potB*), along with repeated mutations in IS4 and ISNCY transposases. While many of the hypothetically-identified missense mutations may contribute to enhanced metabolic or stress response functions, adaptive evolution can also proceed through loss-of-function mutations (Hottes et al., 2013). Ratib et al. analyzed the genomic mutations in *E. coli* under nutrient stress and observed 679 mutations including 147 non-synonymous mutations, and majority of the genes affected are transcriptional regulators, metabolism-related, transporter proteins (Ratib et al., 2021). Several signature genes have also been identified to contribute significantly to antibiotic treatment including *ompR* and *ompF* genes, that encode a transcriptional regulator that controls the responses of microbes to osmotic and acid stress. Although whole-genome sequencing of the bulk populations revealed numerous genetic changes that may be associated with stress adaptation, their precise functional impacts remain uncharacterized. Because these changes can result in either gain or loss of function, integration with transcriptional data is essential to infer biological significance arising from changes in expression. The exploratory nature of this study will facilitate future studiesto explore targeted functional assays (e.g., knockouts, transcriptomic analysis) to validate the roles of the identified mutated genes and assess whether these mutations confer measurable advantages in stress environments. Further investigation into the molecular mechanisms driving these phenotypes such as expression of toxin-antitoxin systems, stringent stress response regulators, and global stress response genes will provide deeper insights into antibiotic persistence biology across diverse pathogens.

## Conclusion and study limitations

This study demonstrates that the *E. coli* and *M. morganii* isolates investigated in this study show adaptability to environmental stressors, including temperature, pH, and antibiotic pressures. The observed strain-specific variation in growth kinetics and persister-like cell formation in the strains investigated here underscores the diversity of stress-response mechanisms within these species. Heat stress influenced increased meropenem persister-like formation in *E. coli* and, to a lesser extent, in *M. morganii*, suggesting that elevated temperatures may precondition cells for antibiotic tolerance in the studied strains.

Whole-genome sequencing of bulk culture-derived DNA revealed that stress-recovered variants acquire both unique and shared mutations affecting genes involved in metabolism, membrane transport, transposable elements and transcriptional regulation, highlighting that phenotypic antibiotic persistence is accompanied by genomic remodeling. These findings provide evidence that environmental stress can drive both phenotypic and genotypic adaptation, either through *de novo* mutations or via enrichment of pre-existing sub-populations, contributing to bacterial survival under antibiotic challenge.

While the study provides valuable insights, one of the limitations is that growth under environmental stress was assessed primarily using OD600-based kinetics and AUC analysis, which may not always directly reflect viable cell counts under extreme environmental stress. Therefore, OD-based measurements is interpreted as indicators of relative growth trends rather than absolute survival. Since it is an exploratory study, it is limited by the small number of strains analyzed (n=2), absence of non-stressed passage control in the WGS workflow and the absence of functional validation (knockouts, complementation, transcriptomic/proteomic) to confirm the functional consequences of the identified genetic changes. Furthermore, laboratory stress conditions may not fully mimic host-associated environments. Future studies integrating non-stressed passage controls, global gene expression studies, and targeted mutagenesis or knockout will be essential to dissect the molecular basis of antibiotic persistence and resistance.

## Data Availability

The data presented in this study are publicly available. The genome has been deposited to the NCBI database (https://www.ncbi.nlm.nih.gov/), accession PRJNA1400651. Further inquiries can be directed to the corresponding author.

## References

[B1] Abban M. K. Ayerakwa E. A. Isawumi A. (2025). Biofilm and surface-motility profiles under polymyxin B stress in multidrug-resistant KAPE pathogens isolated from Ghanaian hospital ICUs. Exp. Biol. Med. 250:10350. doi: 10.3389/ebm.2025.10350 40546280 PMC12180397

[B2] Abiola I. Abass A. Duodu S. Mosi L. (2018). Characterization of culturable airborne bacteria and antibiotic susceptibility profiles of indoor and immediate-outdoor environments of a research institute in Ghana. AAS Open Res. 1:17. doi: 10.12688/aasopenres.12863.2 32259019 PMC7118738

[B3] Balaban N. Helain S. Lewis K. Ackermann M. Aldridge B. Andersson D. I. . (2019). Definitions and guidelines for research on antibiotic persistence. Nat. Rev. Microbiol. 17, 441–448. doi: 10.1038/s41579-019-0196-3 30980069 PMC7136161

[B4] Behera D. U. Dixit S. Gaur M. Mishra R. Sahoo R. K. Sahoo M. . (2023). Sequencing and characterization of m. morganii strain UM869: a comprehensive comparative genomic analysis of virulence, antibiotic resistance, and functional pathways. Genes 14:1279. doi: 10.3390/genes14061279 37372459 PMC10298637

[B5] Carter M. Q. Carychao D. Bono J. L. (2024). Comparative analyses of persistence traits in Escherichia coli O157:H7 strains belonging to different clades including REPEXH01 and REPEXH02 strains. Front. Microbiol. 15:1501956. doi: 10.3389/fmicb.2024.1501956 39744393 PMC11688487

[B6] Cebrián G. Condón S. Mañas P. (2017). Physiology of the inactivation of vegetative bacteria by thermal treatments: mode of action, influence of environmental factors and inactivation kinetics. Foods 6:107. doi: 10.3390/foods6120107 29189748 PMC5742775

[B7] Chen Y-T. Peng H-L. Shia W-C. Hsu F-R. Ken C-F. Tsao Y-M. . (2012). Whole-genome sequencing and identification of Morganella morganii KT pathogenicity-related genes. BMC Genom. 13 (Suppl_7), S4. doi: 10.1186/1471-2164-13-S7-S4 23282187 PMC3521468

[B8] Cho H. Misra R. (2021). Mutational activation of antibiotic-resistant mechanisms in the absence of major drug efflux systems of *Escherichia coli. J. Bacteriol*. 203:e0010921. doi: 10.1128/JB.00109-21 PMC8223954 33972351

[B9] CLSI (2020). Performance Standards for Antimicrobial Susceptibility Testing (CLSI Supplement M100), 30th edition. Malvern, PA: Clinical and Laboratory Standards Institute.

[B10] Dam S. Pagès J.-M. Masi M. (2018). Stress responses, outer membrane permeability control and antimicrobial resistance in Enterobacteriaceae. Microbiology 164, 260–267. doi: 10.1099/mic.0.000613 29458656

[B11] Dash K. K. Fayaz U. Dar A. H. Shams R. Manzoor S. Sundarsingh A. . (2022). A comprehensive review on heat treatments and related impact on the quality and microbial safety of milk and milk-based products. Food Chem. Adv. 1:100041. doi: 10.1016/j.focha.2022.100041

[B12] Dawan J. Ahn J. (2022). Bacterial stress responses as potential targets in overcoming antibiotic resistance. Microorganisms 10:1385. doi: 10.3390/microorganisms10071385 35889104 PMC9322497

[B13] Deatherage D. Barrick J. (2014). Identification of mutations in laboratory-evolved microbes from next-generation sequencing data using breseq. Methods Mol. Biol. 1151, 165-188. doi: 10.1007/978-1-4939-0554-6_12 24838886 PMC4239701

[B14] El-Radhi A. S. M. (2012). Fever management: evidence vs. current practice. World J. Clin. Pediatr. 1, 29–33. doi: 10.5409/wjcp.v1.i4.29 25254165 PMC4145646

[B15] Ewels P. Magnusson M. Lundin S. Käller M. (2016). MultiQC: summarize analysis results for multiple tools and samples in a single report. Bioinformatics 32, 3047–3048. doi: 10.1093/bioinformatics/btw354 27312411 PMC5039924

[B16] Fauvart M. De Groote V. N. Michiels J. (2011). Role of persister cells in chronic infections: clinical relevance and perspectives on anti-persister therapies. J. Med. Microbiol. 60, 699–709. doi: 10.1099/jmm.0.030932-0 21459912

[B17] Fierheller M. Sibbald R. G. (2010). A clinical investigation into the relationship between increased periwound skin temperature and local wound infection in patients with chronic leg ulcers. Adv. Skin Wound Care 23, 369–379. doi: 10.1097/01.ASW.0000383197.28192.98 20631603

[B18] Fisher R. A. Gollan B. Helaine S. (2017). Persistent bacterial infections and persister cells. Nat. Rev. Microbiol. 15, 453–464. doi: 10.1038/nrmicro.2017.42 28529326

[B19] Hall-Stoodley L. Costerton J. Stoodley P. (2004). Bacterial biofilms: from the natural environment to infectious diseases. Nat. Rev. Microbiol. 2:821. doi: 10.1038/nrmicro821 15040259

[B20] Hazan R. Maura D. Que Y. A. Rahme L. G. (2014). Assessing *Pseudomonas aeruginosa* persister/antibiotic tolerant cells. Methods Mol. Biol. 1149, 699–707. doi: 10.1007/978-1-4939-0473-0_54 24818944 PMC6538066

[B21] Hottes A. K. Freddolino P. L. Khare A. Donnell Z. N. Liu J. C. Tavazoie S. . (2013). Bacterial adaptation through loss of function. PLoS Genet. 9:e1003617. doi: 10.1371/journal.pgen.1003617 23874220 PMC3708842

[B22] Hussain A. Bhando T. Casius A. Gupta R. Pathania R. (2025). Deciphering meropenem persistence in *Acinetobacter baumannii* facilitates discovery of anti-persister activity of thymol. Antimicrob. Agents Chemother. 69, e01381–e01324. doi: 10.1128/aac.01381-24 39976427 PMC11963602

[B23] Jiao J. Lv X. Shen C. Morigen M. (2024). 2024. Genome and transcriptomic analysis of the adaptation of Escherichia coli to environmental stresses. Comput. Struct. Biotechnol. J. 23, 2132–2140. doi: 10.1016/j.csbj.2024.05.033 38817967 PMC11137339

[B24] Kamruzzaman M. Iredell J. (2019). A ParDE-family toxin antitoxin system in major resistance plasmids of Enterobacteriaceae confers antibiotic and heat tolerance. Sci. Rep. 9:9872. doi: 10.1038/s41598-019-46318-1 31285520 PMC6614396

[B25] Kuczyńska-Wiśnik D. Stojowska K. Matuszewska E. Leszczyńska D. Algara M. M. Augustynowicz M. . (2015). Lack of intracellular trehalose affects formation of *Escherichia coli* persister cells. Microbiol. Read. Engl. 161, 786–796. doi: 10.1099/mic.0.000012 25500492

[B26] Lee J. S. Choi J-Y. Chung E. S. Peck K. R. Ko K. S. (2019). 2019. Variation in the formation of persister cells against meropenem in Klebsiella pneumoniae bacteremia and analysis of its clinical features. Diagn. Microbiol. Infect. Dis. 95:114853. doi: 10.1016/j.diagmicrobio.2019.06.005 31353067

[B27] Leung V. Lévesque C. M. (2012). A stress-inducible quorum-sensing peptide mediates the formation of persister cells with noninherited multidrug tolerance. J. Bacteriol. 194, 2265–2274. doi: 10.1128/JB.06707-11 22366415 PMC3347057

[B28] Liang J. Cameron G. Faucher S. P. (2023). Development of heat-shock resistance in Legionella pneumophila modeled by experimental evolution. Appl. Environ. Microbiol. 89, e00666–e00623. doi: 10.1128/aem.00666-23 37668382 PMC10537758

[B29] Liu H. Zhu J. Hu Q. Rao X. (2016). 2016. Morganella morganii, a non-negligent opportunistic pathogen. Int. J. Infect. Dis. IJID Off. Publ. Int. Soc. Infect. Dis. 50, 10–17. doi: 10.1016/j.ijid.2016.07.006 27421818

[B30] Long X. Gong Z. Gan Y. Yuan P. Tang Y. Yang Y. . (2024). Sensitive detection of *Escherichia coli* O157:H7 Using allosteric probe and hairpin switches-based isothermal transcription amplification. Anal. Chem. 96, 15608–15613. doi: 10.1021/acs.analchem.4c02413 39307963

[B31] Luber P. Bartelt E. Genschow E. Wagner J. Hahn H. (2003). Comparison of broth microdilution, E Test, and agar dilution methods for antibiotic susceptibility testing of *campylobacter jejuni* and campylobacter coli. J. Clin. Microbiol. 41, 1062–1068. doi: 10.1128/JCM.41.3.1062-1068.2003 12624030 PMC150256

[B32] Mancuso G. Midiri A. Gerace E. Biondo C. (2021). Bacterial antibiotic resistance: the most critical pathogens. Pathogens 10:1310. doi: 10.3390/pathogens10101310 34684258 PMC8541462

[B33] Maurer L. M. Yohannes E. Bondurant S. S. Radmacher M. Slonczewski J. L. (2005). pH regulates genes for flagellar motility, catabolism, and oxidative stress in *Escherichia coli* K-12. J. Bacteriol. 187, 304–319. doi: 10.1128/JB.187.1.304-319.2005 15601715 PMC538838

[B34] Minnullina L. Pudova D. Shagimardanova E. Shigapova L. Sharipova M. Mardanova A. . (2019). Comparative genome analysis of uropathogenic *Morganella morganii* strains. Front. Cell. Infect. Microbiol. 9:167. doi: 10.3389/fcimb.2019.00167 31231616 PMC6558430

[B35] Motta S. S. Cluzel P. Aldana M. (2015). Adaptive resistance in bacteria requires epigenetic inheritance, genetic noise, and cost of efflux pumps. PLoS ONE 10:e0118464. doi: 10.1371/journal.pone.0118464 25781931 PMC4363326

[B36] Niu H. Gu J. Zhang Y. (2024). Bacterial persisters: molecular mechanisms and therapeutic development. Signal Transduct. Target. Ther. 9, 1–32. doi: 10.1038/s41392-024-01866-5 39013893 PMC11252167

[B37] Padan E. Bibi E. Ito M. Krulwich T. A. (2005). 2005. Alkaline pH homeostasis in bacteria: New insights. Biochim. Biophys. Acta BBA - Biomembr. 1717, 67–88. doi: 10.1016/j.bbamem.2005.09.010 16277975 PMC3072713

[B38] Parks D. H. Imelfort M. Skennerton C. T. Hugenholtz P. Tyson G. W. (2015). CheckM: assessing the quality of microbial genomes recovered from isolates, single cells, and metagenomes. Genome Res. 25:1043. doi: 10.1101/gr.186072.114 25977477 PMC4484387

[B39] Paul S. P. Newman L. M. Mubashar Y. Turner P. C. (2005). Morganella morganii: a rare cause of early onset neonatal sepsis and meningitis. Br. J. Hosp. Med. Lond. Engl. 81, 1–3. doi: 10.1101/gr.186072.114 33135916

[B40] Podlesek Z. Bertok D. Ž. (2020). The DNA damage inducible SOS response is a key player in the generation of bacterial persister cells and population wide tolerance. Front. Microbiol. 11:1785. doi: 10.3389/fmicb.2020.01785 32849403 PMC7417476

[B41] Poole K. (2012). Bacterial stress responses as determinants of antimicrobial resistance. J. Antimicrob. Chemother. 67, 2069–2089. doi: 10.1093/jac/dks196 22618862

[B42] Rabkin J. M. Hunt T. K. (1960). Local heat increases blood flow and oxygen tension in wounds. Arch. Surg. Chic. Ill 122, 221–225. doi: 10.1001/archsurg.1987.01400140103014 3813871

[B43] Rahman K. M. T. Amaratunga R. Butzin X. Y. Singh A. Hossain T. Butzin N. C. . (2025). Rethinking dormancy: antibiotic persisters are metabolically active, non-growing cells. Int. J. Antimicrob. Agents 65:107386. doi: 10.1001/archsurg.1987.01400140103014 39551274

[B44] Ratib N. R. Seidl F. Ehrenreich I. M. Finkel S. E. (2021). Evolution in long-term stationary-phase batch culture: emergence of divergent escherichia coli lineages over 1,200 days. mBio 12:e03337. doi: 10.1128/mBio.03337-20 33500336 PMC7858067

[B45] Razmi N. Lazouskaya M. Pajcin I. Petrovic B. Grahovac J. Simic M. . (2023). Monitoring the effect of pH on the growth of pathogenic bacteria using electrical impedance spectroscopy. Results Eng. 20:101425. doi: 10.1016/j.rineng.2023.101425

[B46] Rodríguez-Verdugo A. Tenaillon O. Gaut B. S. (2016). First-Step Mutations during Adaptation Restore the Expression of Hundreds of Genes. Mol. Biol. Evol. 33, 25–39. doi: 10.1093/molbev/msv228 26500250 PMC4693981

[B47] Salam M. A. Al-Amin M. Y. Salam M. T. Pawar J. S. Akhter N. Rabaan A. A. . (2023). Antimicrobial resistance: a growing serious threat for global public health. Healthcare 11:1946. doi: 10.3390/healthcare11131946 37444780 PMC10340576

[B48] Shi H. Chen X. Yao Y. Xu J. (2022). *Morganella morganii:* an unusual analysis of 11 cases of pediatric urinary tract infections. J. Clin. Lab. Anal. 36:e24399. doi: 10.1002/jcla.24399 35349730 PMC9102756

[B49] Sium S. M. A. Goswami B. Chowdhury S. F. Naser S. R. Sarkar M. K. Faruq M. J. . (2025). An insight into the genome-wide analysis of bacterial defense mechanisms in a uropathogenic *Morganella morganii* isolate from Bangladesh. PLoS ONE 20:e0313141. doi: 10.1371/journal.pone.0313141 39847570 PMC11756799

[B50] Stewart B. Rozen D. E. (2012). Genetic variation for antibiotic persistence in *Escherichia coli*. Evol. Int. J. Org. Evol. 66, 933–939. doi: 10.1111/j.1558-5646.2011.01467.x 22380453

[B51] Su M. Tang T. Tang W. Long Y. Wang L. Liu M. . (2023). Astragalus improves intestinal barrier function and immunity by acting on intestinal microbiota to treat T2DM: a research review. Front. Immunol. 14:1243834. doi: 10.3389/fimmu.2023.1243834 37638043 PMC10450032

[B52] Swings T. Van den Bergh B. Wuyts S. Oeyen E. Voordeckers K. Verstrepen K. J. . (2017). Adaptive tuning of mutation rates allows fast response to lethal stress in Escherichia coli. eLife 6:e22939. doi: 10.7554/eLife.22939 28460660 PMC5429094

[B53] Tankeshwar A. (2019). Psychrophiles, Mesophiles, Thermophiles. Microbe Online. Available online at: https://microbeonline.com/psychrophiles-mesophiles-thermophiles/ (Accessed Sepetember 18, 2025).

[B54] Uruén C. Chopo-Escuin G. Tommassen J. Mainar-Jaime R. C. Arenas J. (2020). Biofilms as promoters of bacterial antibiotic resistance and tolerance. Antibiotics 10:3. doi: 10.3390/antibiotics10010003 33374551 PMC7822488

[B55] Vivijs B. Aertsen A. Michiels C. W. (2016). Identification of genes required for growth of *Escherichia coli* MG1655 at moderately low pH. Front. Microbiol. 7:201601672 doi: 10.3389/fmicb.2016.01672 27826291 PMC5078493

[B56] Wißmann J. E. Kirchhoff L. Brüggemann Y. Todt D. Steinmann J. Steinmann E. . (2021). Persistence of pathogens on inanimate surfaces: a narrative review. Microorganisms 9:343. doi: 10.3390/microorganisms9020343 33572303 PMC7916105

[B57] Zhou Y. Liao H. Pei L. Pu Y. (2023). Combatting persister cells: the daunting task in post-antibiotics era. Cell Insight 2:100104. doi: 10.3390/microorganisms9020343 37304393 PMC10250163

[B58] Zhuang H.-H. Qu Q. Long W.-M. Hu Q. Wu X.-L. Chen Y. . (2025). Ceftazidime/avibactam versus polymyxin B in carbapenem-resistant Klebsiella pneumoniae infections: a propensity score-matched multicenter real-world study. Infection 53, 95–106. doi: 10.1016/j.jiph.2023.04.014 38884857 PMC11825550

[B59] Zhuang H-H. Chen Y. Hu Q. Long W,-. M. . (2023). Efficacy and mortality of ceftazidime/avibactam-based regimens in carbapenem-resistant Gram-negative bacteria infections: a retrospective multicenter observational study. J. Infect. Public Health 16, 938–947. doi: 10.1007/s15010-024-02324-8 37087853

