# Supplementary material for: Environmental stress shapes persistence-like phenotypes and genomic changes in Escherichia coli and Morganella morganii: an exploratory study
Source: Front Microbiol. 2026 Mar 17;17:1749211. doi: 10.3389/fmicb.2026.1749211 (PMC13036971; doi:10.3389/fmicb.2026.1749211)
Supplement: Supplementary file 1 [file Supplementary_file_1.docx]

**Supplementary Table 1.** Area under the curve (AUC) of raw growth curve OD600 values for *E. coli* (EC1, EC3, EC4) and *M. morganii* (m5) strains under different stress conditions, including elevated temperature (45 °C), low temperature (4 °C), and varying pH (3, 9, and 10). Control (Ctrl) represents optimal growth conditions (37 °C, pH 6.8). Data are presented as mean values from two independent biological replicates (n = 2)

|  | EC1 | | EC3 | | EC4 | | m5 | |
| --- | --- | --- | --- | --- | --- | --- | --- | --- |
| **Condition** | **AUC** | **SEM** | **AUC** | **SEM** | **AUC** | **SEM** | **M5** | **SEM** |
| 37 °C (Ctrl) | 7.572 | 0.07538 | 7.685 | 0.04991 | 7.066 | 0.05641 | 5.605 | 0.03659 |
| 45 °C | 6.385 | 0.08795 | 5.751 | 0.04792 | 5.231 | 0.0571 | 2.041 | 0.02644 |
| 4 °C | 2.772 | 0.02638 | 2.49 | 0.01809 | 2.212 | 0.02125 | 1.949 | 0.006471 |
| pH 3 | 1.198 | 0.0515 | 0.6743 | 0.003335 | 1.123 | 0.009994 | 1.106 | 0.01699 |
| pH 9 | 0.5638 | 0.02033 | 4.96 | 0.0251 | 2.987 | 0.01078 | 3.575 | 0.02021 |
| pH 10 | 0.9798 | 0.01035 | 0.9505 | 0.007746 | 0.9348 | 0.01011 | 1.365 | 0.008559 |
| Osm | 1.840 | 0.03657 | 1.563 | 0.05833 | 1.512 | 0.05150 | 2.880 | 0.2875 |

SEM – Standard Error of Mean


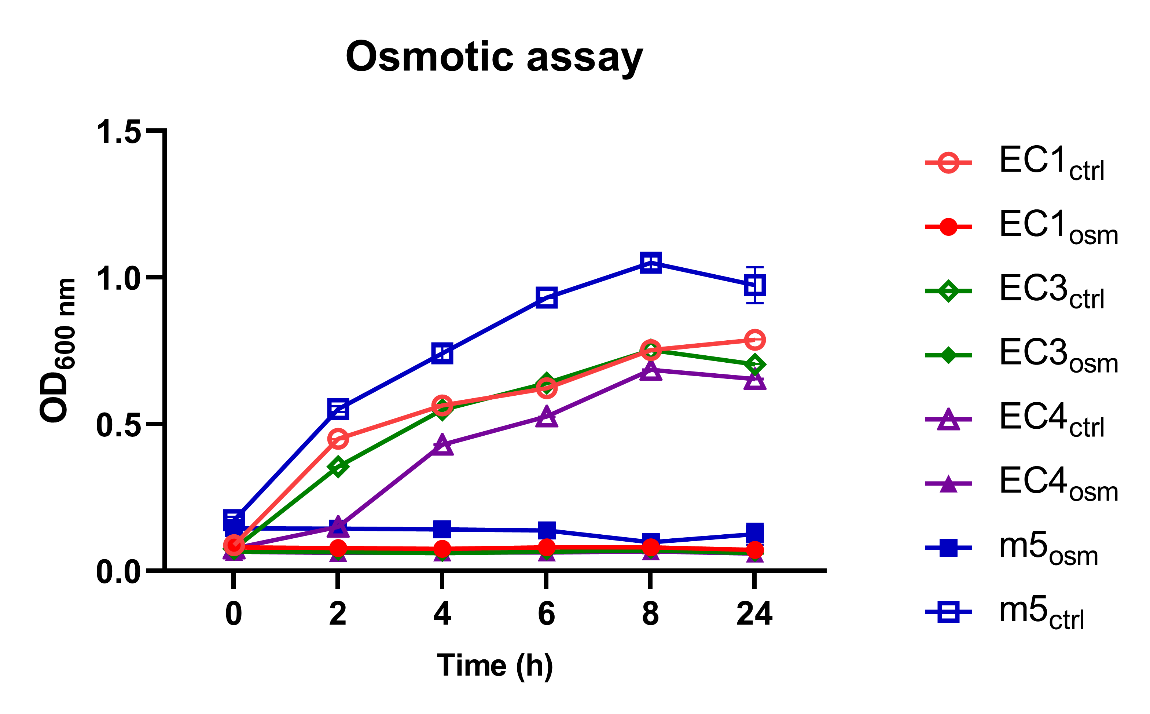


**Supplementary Figure 1.** Growth curves of *E. coli* (EC1, EC3, EC4) and *M. morganii* (m5) strains at OD 600nm under Osmotic stress condition (osm). Control (Ctrl) represents optimal growth conditions. Data are presented as mean ± SEM from two independent biological replicates (n = 2). Due to the limited number of biological replicates, no formal statistical testing was performed, and no significance comparisons are reported. Area under the curve (AUC) was used as a descriptive measure to summarize overall growth trends across conditions (Supplementary Table 1).

**Supplementary Table 2.** Minimum inhibitory concentrations (MIC, µg/mL) and antibiotic susceptibility testing (AST) zone diameters (mm) interpreted according to CLSI guidelines for *E. coli* (EC1, EC3, EC4) and *Morganella morganii* (m5) strains. Values represent the mean of three independent biological replicates (n = 3).

|  |  | **Ciprofloxacin** | |  | | **Meropenem** | |
| --- | --- | --- | --- | --- | --- | --- | --- |
| **Samples** | **AST Zone diameter** | **CLSI**  **Inference** | **MIC values** | **AST Zone diameter** | **Inference** | | **MIC values** |
| EC1 | - | Resistant | 640 µg/mL | 19.5 mm | Resistant | | 10 µg/mL |
| EC3 | - | Resistant | 640 µg/mL | 19 mm | Resistant | | 10 µg/mL |
| EC4 | - | Resistant | 320 µg/mL | 20.5 mm | Intermediate | | 10 µg/mL |
| m5 | - | Resistant | 320 µg/mL | 21 mm | Intermediate | | 10 µg/mL |

**Supplementary Table 3.** Colony-forming units (CFU/mL) of persister-like subpopulations for *E. coli* (EC1, EC3, EC4) and *Morganella morganii* (m5) strains under antibiotic treatments; A) Meropenem and B) Ciprofloxacin. Initial samples collected prior to antibiotic exposure (Ini) served as untreated controls and represent the starting inoculum. Duplicate biological replicates (n = 2) are shown. CFU/mL was calculated based on serial dilutions and plating as described in the Methods section.

|  | **m5** | | **EC1** | | **EC3** | | **EC4** | |
| --- | --- | --- | --- | --- | --- | --- | --- | --- |
| Time  (h) | Replicate 1  (CFU/mL) | Replicate 2  (CFU/mL) | Replicate 1  (CFU/mL) | Replicate 2  (CFU/mL) | Replicate 1  (CFU/mL) | Replicate 2  (CFU/mL) | Replicate 1  (CFU/mL) | Replicate 2  (CFU/mL) |
| Initial | 1.08 × 10^10^ | 1.36 × 10^11^ | 7 × 10^7^ | 2.024 × 10^7^ | 1.18 × 10^10^ | 9.1 × 10^10^ | 3.8 × 10^10^ | 2.34 × 10^11^ |
| 1 | 1.1 × 10^5^ | 5.0 × 10^4^ | 3.4 × 10^5^ | 3.4 × 10^5^ | 7.0 × 10^5^ | 3.0 × 10^5^ | 6.5 × 10^5^ | 4.5 × 10^5^ |
| 3 | 1.6 × 10^4^ | 1.0 × 10^4^ | 2.7 × 10^5^ | 2.24 × 10^5^ | 3.0 × 10^4^ | 3.0 × 10^4^ | 4.0 × 10^4^ | 7.0 × 10^4^ |
| 5 | 1.0 × 10^3^ | 5.0 × 10^3^ | 2.0 × 10^4^ | 2.0 × 10^4^ | 1.0 × 10^3^ | 1.0 × 10^3^ | 2.0 × 10^3^ | 1.0 × 10^3^ |

1. **Meropenem**

|  | **m5** | | **EC1** | | **EC3** | | **EC4** | |
| --- | --- | --- | --- | --- | --- | --- | --- | --- |
| Time  (h) | Replicate 1  (CFU/mL) | Replicate 2  (CFU/mL) | Replicate 1  (CFU/mL) | Replicate 2  (CFU/mL) | Replicate 1  (CFU/mL) | Replicate 2  (CFU/mL) | Replicate 1  (CFU/mL) | Replicate 2  (CFU/mL) |
| Initial | 5.8 × 10^10^ | 5.5 × 10^10^ | 7.0 × 10^10^ | 6.0 × 10^9^ | 1.2 × 10^10^ | 9.1 × 10^10^ | 3.8 × 10^10^ | 2.34 × 10^11^ |
| 1 | 1.8 × 10^7^ | 1.6× 10^7^ | 1.84 × 10^5^ | 3.4 × 10^5^ | 1.1 × 10^6^ | 7.0 × 10^5^ | 1.5 × 10^7^ | 4.1 × 10^7^ |
| 3 | 1.1 × 10^5^ | 5.0 × 10^4^ | 2.8 × 10^5^ | 3.6 × 10^5^ | 7.2 × 10^3^ | 1.4 × 10^4^ | 3.2 × 10^6^ | 1.8 × 10^6^ |
| 5 | 1.6 × 10^4^ | 1.0 × 10^4^ | 3.0 × 10^4^ | 2.0 × 10^4^ | 5.2 × 10^3^ | 1.0 × 10^4^ | 2.3 × 10^5^ | 1.4 × 10^5^ |

1. **Ciprofloxacin**

**Supplementary Table 4.** Genome mapping metrics of parental variants in *E. coli and M. morganii* parental (EC1, EC3, m5) and stress-recovered variants, including (cip, ciprofloxacin recovered; osm, osmotic stress recovered; ph9, alkaline stress recovered; T45, temperature at 45 °C recovered)

| **Isolate** | **Genome length (bp)** | **Coverage bases** | **Genome Coverage** | **Mean_MAPQ (mean mapping quality)** |
| --- | --- | --- | --- | --- |
| EC1 | 5198239 | 4048818 | 87.22 | 59.2 |
| Cip_EC1 | 5131675 | 4031502 | 86.85 | 59.16 |
| T45_EC1 | 5239383 | 4054216 | 87.34 | 59.2 |
| Osm_EC1 | 5222049 | 4009320 | 86.37 | 59.2 |
| EC3 | 5120222 | 4027800 | 86.77 | 59.2 |
| Cip_EC3 | 5132774 | 4032921 | 86.88 | 59.1 |
| T45_EC3 | 5123581 | 4029056 | 86.80 | 59.2 |
| Osm_EC3 | 5202931 | 4028028 | 86.78 | 59.2 |
| m5 | 3991362 | 3633490 | 93.40 | 59.3 |
| Cip_m5 | 3997342 | 3632668 | 93.38 | 59.2 |
| T45_m5 | 3991601 | 3632586 | 93.38 | 59.2 |
| Ph9_m5 | 3992320 | 3636633 | 93.48 | 59.1 |
| Osm_m5 | 3993899 | 3633599 | 93.41 | 59.2 |

**Supplementary Table 5.** BioSample accession numbers for the isolates

**BioSample Accession Sample Name**

SAMN54541775 EC1

SAMN54541776 Cip_EC1

SAMN54541777 Osm_EC1

SAMN54541778 T45_EC1

SAMN54541779 EC3

SAMN54541780 Cip_EC3

SAMN54541781 Osm_EC3

SAMN54541782 T45_EC3

SAMN54541783 EC4

SAMN54541784 m5

SAMN54541785 Cip_m5

SAMN54541786 T45_m5

SAMN54541787 Osm_m5

SAMN54541788 Ph9_m5
